# Myocardial T_1_ and T_2_ mapping at 3 T: reference values, influencing factors and implications

**DOI:** 10.1186/1532-429X-15-53

**Published:** 2013-06-18

**Authors:** Florian von Knobelsdorff-Brenkenhoff, Marcel Prothmann, Matthias A Dieringer, Ralf Wassmuth, Andreas Greiser, Carsten Schwenke, Thoralf Niendorf, Jeanette Schulz-Menger

**Affiliations:** 1 Berlin Ultrahigh Field Facility, Max-Delbrueck Center for Molecular Medicine, Berlin, Germany; 2 Working Group on Cardiovascular Magnetic Resonance, Experimental and Clinical Research Center a joint cooperation between the Charité Medical Faculty and the Max-Delbrueck Center for Molecular Medicine HELIOS Klinikum Berlin Buch, Department of Cardiology and Nephrology, Lindenberger Weg 80, 13125, Berlin, Germany; 3 Siemens Healthcare, Erlangen, Germany; 4 Scossis, Berlin, Germany; 5 Experimental and Clinical Research Center, a joint cooperation between the Charité Medical Faculty and the Max-Delbrueck Center for Molecular Medicine, Berlin, Germany

**Keywords:** Cardiovascular magnetic resonance, Heart, T_1_, T_2_, Mapping, 3 T

## Abstract

**Background:**

Myocardial T_1_ and T_2_ mapping using cardiovascular magnetic resonance (CMR) are promising to improve tissue characterization and early disease detection. This study aimed at analyzing the feasibility of T_1_ and T_2_ mapping at 3 T and providing reference values.

**Methods:**

Sixty healthy volunteers (30 males/females, each 20 from 20–39 years, 40–59 years, 60–80 years) underwent left-ventricular T_1_ and T_2_ mapping in 3 short-axis slices at 3 T. For T_2_ mapping, 3 single-shot steady-state free precession (SSFP) images with different T_2_ preparation times were acquired. For T_1_ mapping, modified Look-Locker inversion recovery technique with 11 single shot SSFP images was used before and after injection of gadolinium contrast. T_1_ and T_2_ relaxation times were quantified for each slice and each myocardial segment.

**Results:**

Mean T_2_ and T_1_ (pre-/post-contrast) times were: 44.1 ms/1157.1 ms/427.3 ms (base), 45.1 ms/1158.7 ms/411.2 ms (middle), 46.9 ms/1180.6 ms/399.7 ms (apex). T_2_ and pre-contrast T_1_ increased from base to apex, post-contrast T_1_ decreased. Relevant inter-subject variability was apparent (scatter factor 1.08/1.05/1.11 for T_2_/pre-contrast T_1_/post-contrast T_1_). T_2_ and post-contrast T_1_ were influenced by heart rate (p < 0.0001, p = 0.0020), pre-contrast T_1_ by age (p < 0.0001). Inter- and intra-observer agreement of T_2_ (r = 0.95; r = 0.95) and T_1_ (r = 0.91; r = 0.93) were high. T_2_ maps: 97.7% of all segments were diagnostic and 2.3% were excluded (susceptibility artifact). T_1_ maps (pre-/post-contrast): 91.6%/93.9% were diagnostic, 8.4%/6.1% were excluded (predominantly susceptibility artifact 7.7%/3.2%).

**Conclusions:**

Myocardial T_2_ and T_1_ reference values for the specific CMR setting are provided. The diagnostic impact of the high inter-subject variability of T_2_ and T_1_ relaxation times requires further investigation.

## Background

Cardiovascular magnetic resonance (CMR) provides techniques for non-invasive myocardial tissue characterization. T_1_ and T_2_ mapping of the left ventricular myocardium, i.e. quantification of the myocardial T_1_ and T_2_ relaxation times, as well as the T_1_-derived extracellular volume fraction have been demonstrated to add valuable information [1-6]. Most of the experience with myocardial mapping was gained at a magnetic field strength of 1.5 T. Parametric myocardial mapping at 3 T is conceptually appealing due to the signal gain inherent to higher fields, which may be exploited for improved spatial and temporal resolution [7]. Many of the previous studies focused on intra-individual comparison of diseased and remote myocardium. However, T_2_ and T_1_ reference values of all myocardial segments may be important to define small focal abnormalities and to identify diffuse tissue changes in the absence of healthy “remote” myocardium. For all these reasons this study scrutinizes myocardial T_1_ and T_2_ at 3 T in a large sample of healthy volunteers using state-of-the art mapping techniques.

## Methods

### Study population

60 healthy volunteers were enrolled into the study (30 men/30 women, equally distributed within 3 age categories (Table 1)). The status “healthy” was based on: i) uneventful medical history, ii) absence of any symptoms indicating cardiovascular dysfunction, iii) normal ECG, iv) normal cardiac dimensions and function proven by cine CMR. v) normal myocardial tissue assessed by late enhancement (LGE). For each volunteer written informed consent was obtained prior to the study, after due approval by the ethical committee of the Charité Medical Faculty (EA2/077/10). All experiments were performed in compliance with the Helsinki Declaration.

**Table 1:** Characteristics of the volunteers

**Parameter**	**Result**
Number	60
Females/Males	30/30
Age [years]	48 ± 17
Age group 20–39 years	20 (10 Males/10 Females)
Age group 40–59 years	20 (10 Males/10 Females)
Age group 60–80 years	20 (10 Males/10 Females)
Height [cm]	173 ± 9
Weight [kg]	76 ± 14
Body mass index [kg/m^2^]	25 ± 4
Body surface area [m^2^]	1.9 ± 0.2
Systolic blood pressure [mm/Hg]	132 ± 12
Diastolic blood pressure [mm/Hg]	72 ± 11
Heart rate [min^-1^]	70 ± 6
LV enddiastolic volume [ml]	143 ± 35
LV enddiastolic volume index [ml/cm]	0.8 ± 0.2
LV ejection fraction [%]	64 ± 5
LV mass [mg]	101 ± 26
LV mass index [mg/cm]	0.6 ±0.2

### CMR examination

**Figure 1:**
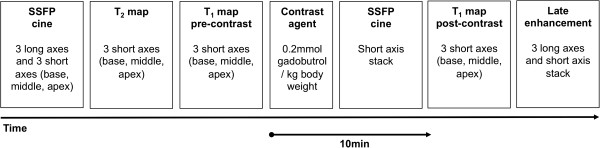
**CMR protocol.** This schematic diagram illustrates the chronological sequence of the applied CMR techniques.

All CMR exams were performed with a 3 T system (Magnetom Verio, Siemens Healthcare, Erlangen, Germany) using a 32-channel cardiac RF coil for signal reception, the integrated body RF coil for transmission, and ECG for cardiac gating. Subject-specific, volume-selective first- and second-order B_0_-shimming based on field maps derived from double-gradient-echo acquisitions was performed to improve static field uniformity. The following CMR protocols were used (Figure 1).

#### Cine imaging

Steady-state free-precession (SSFP) cine images were obtained during repeated breath-holds in three long axes (horizontal, vertical, and 3-chamber) and in a stack of short axes (SAX) covering the left ventricle (LV) to assess wall motion and for cardiac chamber quantification. Imaging parameters were: repetition time (TR) 3.1 ms, echo time (TE) 1.3 ms, asymmetric echo with factor 0.29, flip angle (FA) 45°, field of view (FOV) (276 × 340)mm^2^, matrix 156 × 192, slice thickness 6 mm, receiver bandwidth (BW) 704Hz/px, parallel imaging using GRAPPA reconstruction (R = 2), 30 cardiac phases.

#### T_2_ mapping

For T_2_ mapping, data were acquired in basal, mid-ventricular, and apical SAX planes using a T_2_-prepared single-shot SSFP technique similar to the one described for 1.5 T [2]. For the application on a 3 T platform, the RF pulse length of the SSFP readout module was increased to reduce the SAR deposition and adiabatic T_2_ preparation pulses were employed to improve the homogeneity of the T_2_ weighting. Three SSFP images, each with different T_2_ preparation time (TE_T2P_ = 0 ms, 24 ms, 55 ms) were acquired in end-diastole within one breath-hold. Imaging parameters were: TR = 2.4 ms, TE = 1 ms, FA = 70°, FOV = (340 × 278) mm^2^, matrix = 176 × 144, slice thickness = 6 mm, BW = 1093Hz/px, GRAPPA acceleration factor 2, linear phase encoding scheme. To correct for residual cardiac and respiratory motion between image sets, a non-rigid registration algorithm was used [8]. A pixel-wise myocardial T_2_-map was generated using unsupervised curve-fitting based on a two-parameter equation [2]. The single shot SSFP readout and use of only three TE_T2P_ was chosen to balance accuracy and acquisition time (7 heart cycles) [2,9].

#### T_1_ mapping

For T_1_ mapping, data were acquired in basal, mid-ventricular, and apical SAX planes before and 10 minutes after administration of 0.2 mmol/kg i.v. gadobutrol (Gadovist®, Bayer Healthcare Germany). Data were obtained in end-diastole using a cardiac-gated, SSFP-based Modified Look-Locker Inversion Recovery (MOLLI) technique [10]. For the application at 3 T, the RF pulse length of the SSFP readout module was increased to reduce the SAR deposition. Imaging parameters were: TR = 2.6-2.7 ms, TE = 1.0-1.1 ms, FA = 35°, FOV = (270 × 360)mm^2^, matrix = 156 × 208 to 168 × 224, slice thickness = 6 mm, BW = 1045-1028Hz/px, GRAPPA acceleration factor 2, linear phase-encoding ordering, minimum TI of 91 ms. To generate a pixel-wise myocardial T_1_-map, single-shot SSFP images were acquired at different inversion times (pattern 3-3-5, [10]) and registered [8] prior to a non-linear least-square curve fitting using S(TI) = A - B exp(−TI/T1*) with T1 = T1 × (B/A - 1), where A, B, and T1* are estimated by a three parameter fit [11]. In-plane voxel dimensions were kept isotropic to ensure that partial volume effects are independent of slice rotation.

#### LGE imaging

LGE imaging was performed in the same planes as SSFP CINE imaging using a segmented inversion-recovery gradient-echo sequence beginning 15 minutes after contrast administration. The inversion time (TI) was repeatedly adjusted to appropriately null the myocardium during the length of LGE image acquisition. Imaging parameters were: TR = 10.5 ms, TE = 5.4 ms, FA = 30°, FOV (350 × 262) mm^2^, matrix 256 × 162, slice thickness 6 mm, BW 140Hz/px, GRAPPA acceleration factor 2.

### CMR image analysis

Image analysis was done using CMR^42^ (Circle Cardiovascular Imaging, Calgary, Canada).

#### LV chamber quantification

SSFP cine images were visually evaluated regarding wall motion abnormalities. LV enddiastolic and endsystolic volume and LV mass were determined by manually contouring the endocardial and epicardial borders of the SAX in systole and diastole.

#### LGE assessment

The absence of LGE was determined qualitatively by visual assessment.

#### T_2_ and T_1_ mapping - qualitative assessment

Each single original image was assessed regarding artifacts caused by susceptibility effects, cardiac or respiratory motion. Each motion-corrected series was evaluated whether the images were correctly aligned. Each map was evaluated whether the original images were transformed to a reasonably appearing map. The presence of artifacts led to the exclusion of all affected myocardial segments. Two experienced readers assessed quality in consensus.

#### T_2_ mapping - quantitative assessment

The LV myocardium was delineated by manually contouring the endocardial and epicardial border. We ensured that the region of interest (ROI) was definitely within the myocardium and did not include blood or epicardial fat based. An endocardial and epicardial contour was drawn in one original motion-corrected image. The trabeculated layer and the epicardial border were left out. In doubt, SSFP cine images were consulted. The contours were copied to the other images and adapted to fit in all of these. These final contours were copied to the map. The myocardial ROI was automatically segmented according to the AHA segment model [12]. Results are presented both per segment and averaged per slice.

#### T_1_ mapping - quantitative assessment

T_1_ values were recorded from pre-contrast and post-contrast T_1_ maps applying the same procedure as for T_2_.

#### T_2_ and T_1_ mapping - Observer dependency

Intra- and interobserver variability were tested in a subgroup of 20 randomly selected subjects (320 myocardial segments), where one observer measured T_2_ and pre-contrast T_1_ values of each LV segment twice with at least 3 months of time between the measurements. A second observer measured T_2_ and pre-contrast T_1_ values blinded to the other results.

### Statistical analysis

Baseline characteristics are shown as means with standard deviation (SD) or absolute frequencies. Relaxation times are displayed as least-square means with 95% tolerance intervals (90% coverage) and were assessed by slice and by segment using mixed linear models on logarithmic transformed data to ensure normal distributed data. The following co-factors were included into each model to assess their impact on the relaxation times: age (categories), gender, heart rate (binary with split at median), blood pressure and excluded backwards if not significant. For T_1_ and T_2_, the scatter factor was provided as back transformed SD, which allows a similar interpretation as the coefficient of variation for non-transformed data. All values presented were back transformed using the exponential to present the data on the original scale. Spearman's correlation coefficients were calculated to evaluate correlations between the co-factors, which may interfere with the modelling. A p-value of less than 0.05 was regarded as statistically significant. Calculations were performed using SAS 9.2 (SAS Institute Inc., Cary, NC, USA). Intra- and inter-observer dependency was assessed by Bland-Altman analysis and Pearson’s correlation using Prism 5.0 (Graphpad Software, La Jolla, CA, USA).

## Results

### CMR

All 60 CMR scans were performed without major adverse events. The scans were incomplete in 4 subjects. T_2_ maps were available for 58 subjects, pre-contrast T_1_ maps for 59 subjects and post-contrast T_1_ maps for 57 subjects.

### T_2_ mapping

**Figure 2:**
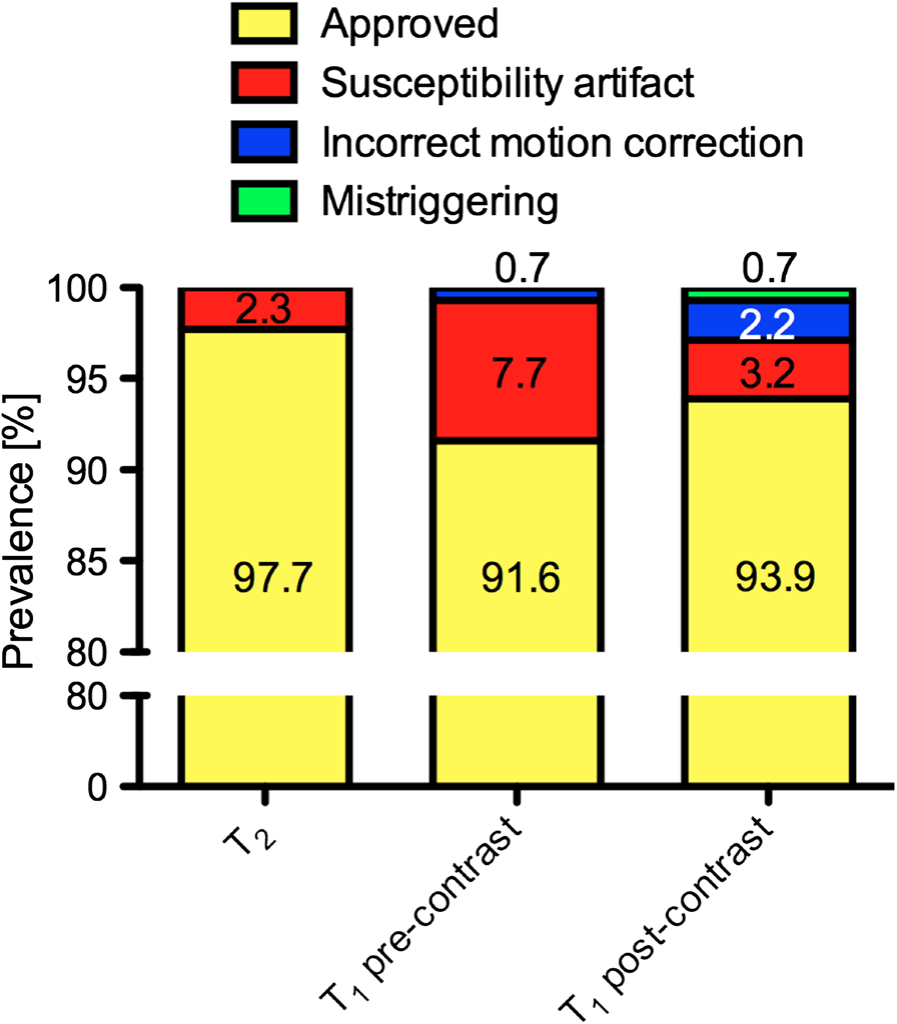
**Quality assessment of T**_**2 **_**and T**_**1 **_**maps.** The bar graphs show the prevalence of images approved as evaluable, as well as the frequency of susceptibility artifacts, incorrect motion correction, and mistriggering for each technique.

**Figure 3:**
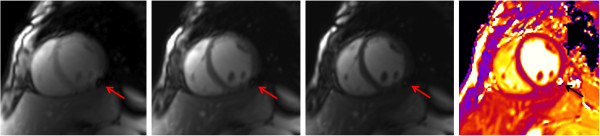
**T**_**2 **_**mapping artifact.** Susceptibility artifact in the inferolateral wall of the midventricular plane (red arrow; the 3 grayscale images represent the T_2_-prepared SSFP images with different T_2_ preparation times). In the map (right image), the artifact is visible in the same area (black arrow).

From 922 segments, 901 (97.7%) were eligible for analysis (Figure 2). A full set of original data and corresponding maps is available as additional file (see Additional file 1). Twenty-one segments (2.3%) were excluded due to a susceptibility artifact (Figures 2 and 3) mainly in the inferior/inferolateral wall (18 out of 21; 85.7%). Exclusion of at least one segment affected 12 out of 58 subjects (20.7%).

T_2_ relaxation times per slice are shown in Table 2. Mean value was 44.1 ms (base), 45.1 ms (middle) and 46.9 ms (apex). All slices differed significantly (p < 0.0001) with increasing values from base to apex.

**Table 2:** **Myocardial T**_**2 **_**and T**_**1 **_**relaxation times [in ms] for each plane (base, middle, apex)**

	**Position**	**Least square mean**	**95% Tolerance interval**	**Min-Max**
T_2_ [ms]	Base	44.1	39.3 – 49.5	36.2 – 53.3
	Middle	45.1	39.9 – 50.1	37.9 – 57.0
	Apex	46.9	40.8 – 53.8	39.1 – 59.1
T_1_ pre-contrast [ms]	Base	1157.1	1074.5 – 1246.0	965.6 – 1340.8
	Middle	1158.7	1074.0 – 1250.1	1005.3 – 1295.9
	Apex	1180.6	1073.9 – 1297.9	1106.3 – 1393.9
T_1_ post-contrast [ms]	Base	427.3	363.2 – 502.7	284.5 – 520.1
	Middle	411.2	349.9 – 483.2	282.5 – 513.2
	Apex	399.7	323.0 – 494.6	260.6 – 519.5

**Figure 4:**
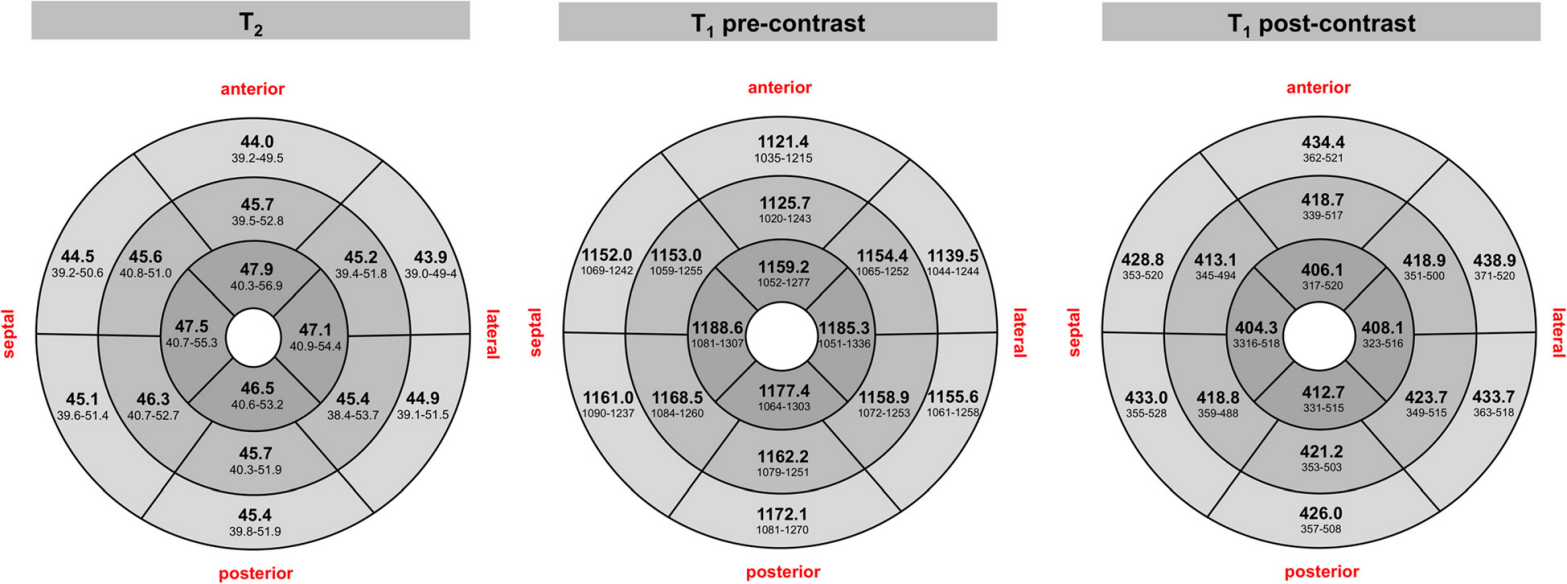
**Mean T**_**2 **_**and T**_**1 **_**relaxation times.** T_2_ and T_1_ relaxation times (ms) for each myocardial segment illustrated as a bulls eye plot that represents the 16 segments of the basal (outer ring), midventricular (middle ring) and apical (central ring) short-axis plane [12]. Results are given as least-square mean and 95% tolerance interval.

**Figure 5:**
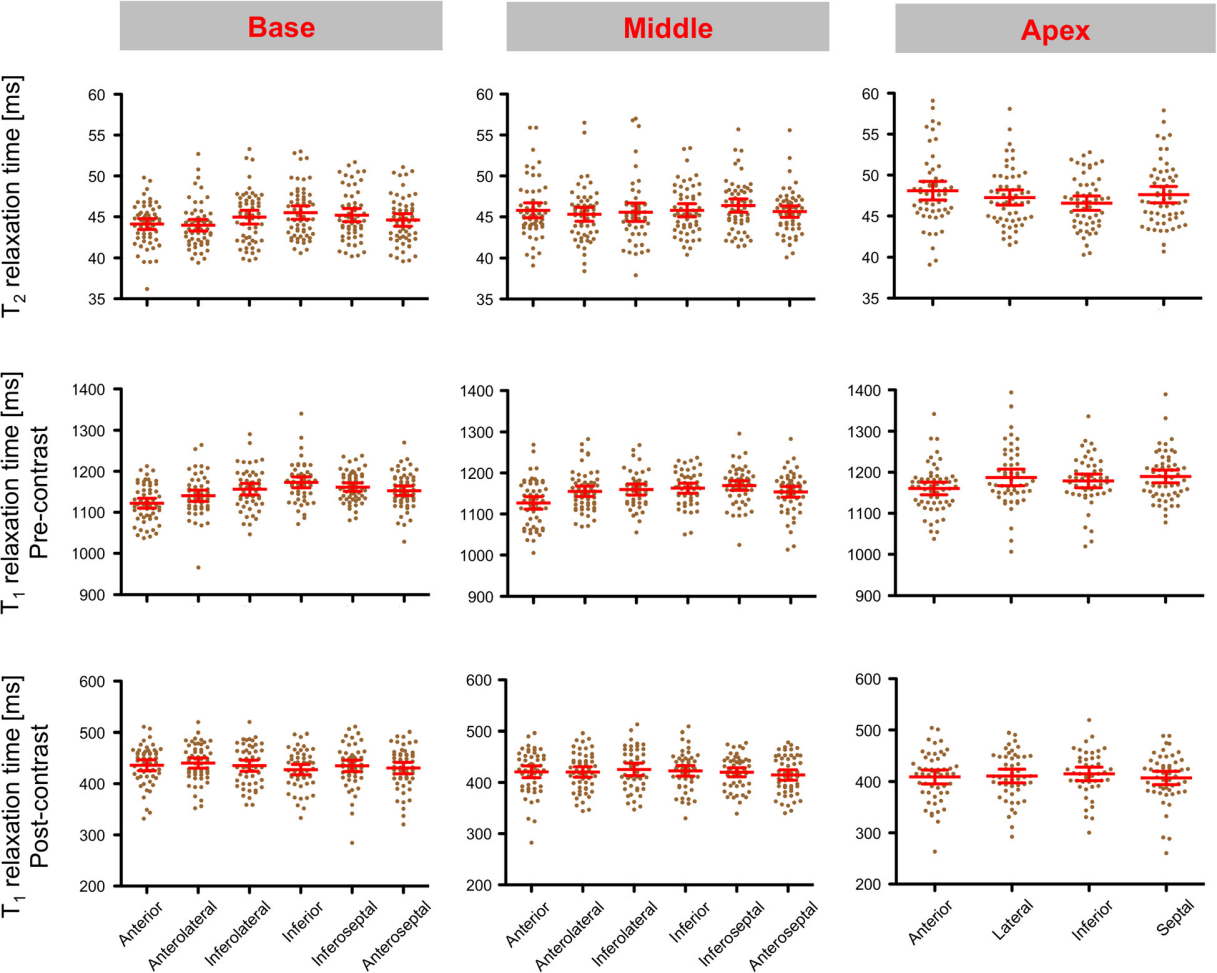
**Individual T**_**2 **_**and T**_**1 **_**relaxation times.** Distribution of all single individual T_2_ and T_1_ relaxation times in each myocardial segment. The red lines indicate the least-square mean and the 95% confidence interval.

T_2_ values for each myocardial segment are presented in Figure 4. Significant segment-to-segment differences were observed in the basal slice (p = 0.0036) with slightly lower values in the anterior wall compared to inferior. No significant segment-to-segment differences were found for the midventricular (p = 0.5398) and apical slices (p = 0.1367). The distribution of all individual T_2_ results is illustrated in Figure 5. A relevant inter-subject variability was evident as indicated by a scatter factor of 1.08 (Figure 5 and Table 2).

Heart rate (ranging from 47 to 102 min^-1^) was found to significantly influence T_2_ measurements (p < 0.0001). A heart rate higher than the median (69.5 min^-1^) was associated with lower T_2_ values (base: 42.8 ms vs. 45.8 ms; middle: 43.9 ms vs. 46.5 ms; apex: 45.7 ms vs. 48.2 ms). Other tested cofactors including age and gender were not found to be significant.

### Pre-contrast T_1_ mapping

**Figure 6:**
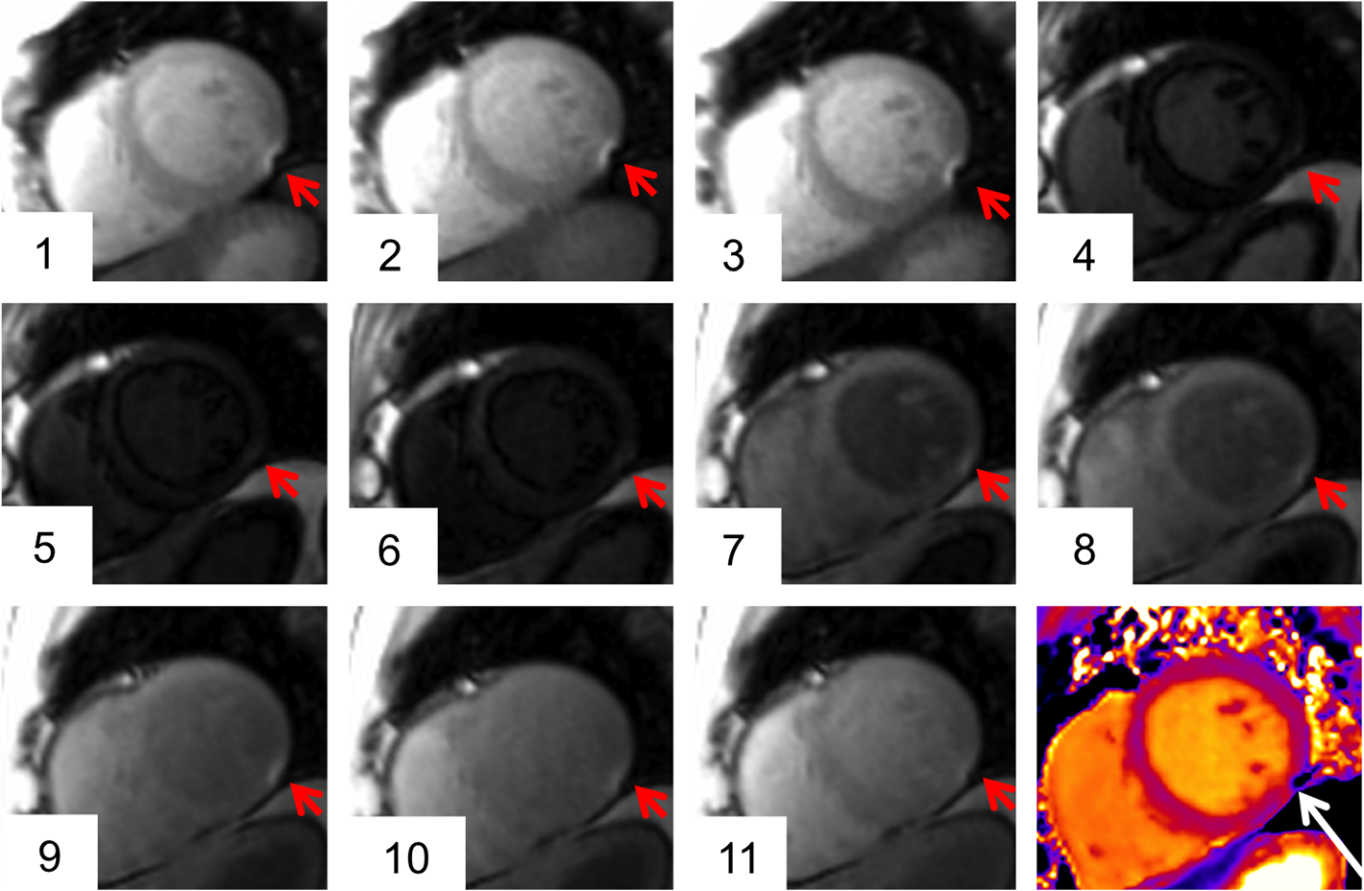
**T**_**1 **_**mapping artifact.** Susceptibility artifact in the inferolateral wall of the midventricular plane (red arrow). The artifact was located at the border of the inferolateral and the inferior segment. In the corresponding map the artifact is hardly recognizable by visual assessment (white arrow).

**Figure 7:**
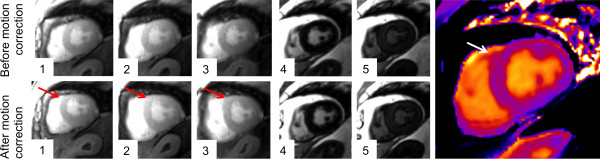
**Failed motion correction during T**_**1 **_**mapping.** The original images (upper row) show the regular shape of the LV myocardium (the first five out of eleven images of the complete T_1_ acquisition are depicted). The motion correction algorithm led to an outbound shift of the anterior and anteroseptal myocardial segment (red arrow in the bottom row). The corresponding map (right image) indicates an inhomogeneous T_1_ distribution in this area (white arrow).

For pre-contrast T_1_ mapping 938 segments were obtained. 859 (91.6%) were eligible for analysis (Figure 2). A full set of original data and corresponding maps is available as additional file (see Additional file 2). Seventy-two segments (7.7%) were excluded due to a susceptibility artifact and 7 segments (0.7%) due to incorrect motion correction (Figures 2, 6 and 7). In 63 out of 72 segments (87.5%) with susceptibility artifact, the inferior/inferolateral segments were affected. Exclusion of at least one segment affected 34 out of 59 subjects (57.6%).

T_1_ relaxation times per slice are shown in Table 2. Mean value was 1157.1 ms (base), 1158.7 ms (middle) and 1180.6 ms (apex). Apical T_1_ relaxation times were significantly larger than basal and midventricular (each p < 0.0001).

T_1_ values for each myocardial segment are shown in Figure 4. A significant segment-to-segment difference was found for each slice (basal: p < 0.0001; mid: p < 0.0001; apex: p = 0.0153). T_1_ of the anterior segment was lower than in the other segments. The distribution of all individual T_1_ results is illustrated in Figure 5. A relevant inter-subject variability was found with a scatter factor of 1.05 (Figure 5, Table 2).

**Figure 8:**
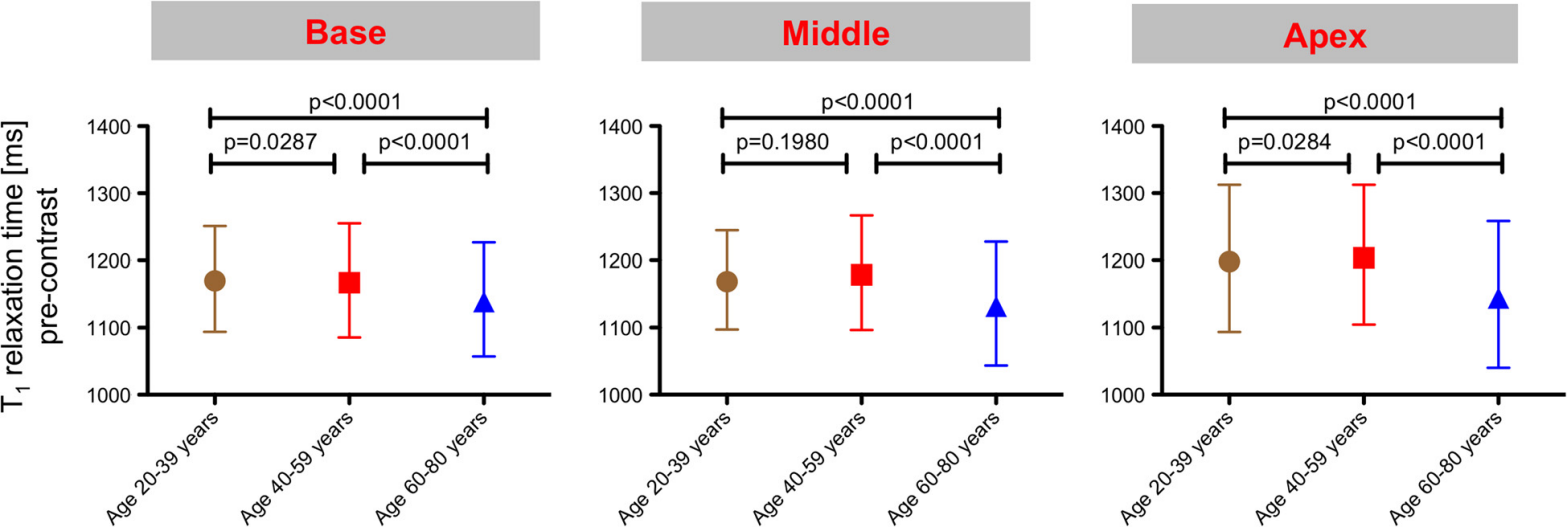
**Age-dependency of T**_**1 **_**times.** Myocardial pre-contrast T_1_ relaxation times grouped by age categories for each slice. Results are given as mean and 95% tolerance interval.

The age categories were found to significantly influence myocardial T_1_ relaxation times (p < 0.0001). The difference was small between age category 20–39 years and 40–59 years. A clear decrease of T_1_ relaxation times was observed for subjects ≥ 60 years (Figure 8). Other tested cofactors including heart rate and gender were not found to be significant.

### Post-contrast T_1_ mapping

For post-contrast T_1_ mapping 841 out of 896 segments (93.9%) were eligible for analysis (Figure 2). Twenty-nine segments (3.2%) were excluded due to a susceptibility artifact, which mainly affected the inferior/inferolateral segments (25 out of 29; 86.2%). Six segments (0.7%) were excluded due to mistriggering (all in one subject). Motion correction failed in one subject in all planes and in one subject in the apical plane leading to an exclusion of 20 segments (2.2%). Exclusion of at least one segment affected 18 out of 56 subjects (32.1%).

T_1_ relaxation times per slice are shown in Table 2. Mean values of 427.3 ms (base), 411.2 ms (middle) and 399.7 ms (apex) were obtained. All slices differed significantly from each other (base vs. middle: p < 0.0001; base vs. apex p < 0.0001; middle vs. apex: p = 0.0013) with decreasing T_1_ values from base to apex.

T_1_ values for each myocardial segment are shown in Figure 4. No significant segment-to-segment differences were observed for each slice (basal: p = 0.4918; mid: p = 0.4741; apex: p = 0.5629). The distribution of all individual T_1_ results is illustrated in Figure 5. Post-contrast T_1_-maps revealed a relevant inter-subject variability reflected by a scatter factor of 1.11 (Figure 5, Table 2).

Heart rate was found to significantly influence the post-contrast T_1_ relaxation time (p = 0.0020) with higher heart rates than the median (69.5 bpm) being associated with lower post-contrast T_1_ relaxation times (base: 445.7 ms vs. 418.7 ms; middle: 430.1 ms vs. 405.3 ms; apex: 427.6 ms vs. 388.0 ms). Other tested cofactors including age and gender were not found to be significant.

For T_2_ and pre-contrast T_1_ mapping inter- and intra-observer analysis demonstrated close agreement (Table 3).

**Table 3:** **Intra- and inter-observer dependency of the segmental quantification of T**_**2 **_**and pre-contrast T**_**1 **_**relaxation times**

**Technique**	**Correlation coefficient r**	**Bland-Altman: Bias ± SD [ms]**
T_2_ – Intra-observer	0.95	0.0 ± 1.3
T_2_ – Inter-observer	0.95	0.1 ± 1.1
T_1_ – Intra-observer	0.93	4.6 ± 18.3
T_1_ – Inter-observer	0.91	0.5 ± 20.2

## Discussion

This study examined myocardial T_1_ and T_2_ mapping techniques at 3 T in a large sample of healthy volunteers. The main findings are: i) T_2_ and T_1_ mapping achieve a high grade of diagnostic image quality, although susceptibility artifacts entailed the exclusion of a limited number of myocardial segments from the analysis. ii) Observer dependency of T_2_ and T_1_ relaxation time quantification was low. iii) Mean values and 95% tolerance interval of myocardial T_2_ and T_1_ relaxation times are presented per slice and per segment and can be used as reference values specific for this MR setting.iii) An inter-subject distribution of T_2_ and T_1_ values became apparent and may constitute a limitation to define appropriate cut-offs.

### T_2_ mapping

Previous studies with SSFP-based T_2_ mapping at 1.5T did not report the exclusion of segments from analysis due to SSFP off-resonance or banding artifacts [2-4]. Hence, this challenge seems to surface at higher field strengths due to the increase in the peak-to-peak B_0_ inhomogeneity across the heart. The use of an appropriately selected delta frequency may be an option to resolve some artifacts and deserves further systematic investigation. The artifacts mainly affected the inferolateral region, where pathologies like myocarditis may also exhibit their predominant lesion [13]. Despite that, the step from 1.5 T to 3 T for CMR is generally desired due to expected gains in signal, which may be exploited for improved spatial and temporal resolution. This potential promises to enable more detailed insights into cardiac tissue in order to facilitate the early detection of myocardial disease.

T_2_ relaxation times derived from T_2_-prepared SSFP imaging in this study are higher compared to a black-blood multi-echo spin-echo approach at 3 T, which provided a mean value of T_2_ = 39.6m sin the septum [14]. Myocardial T_2_ reported here was found to be lower versus a mean T_2_ = 52.2 ms reported for T2 prepared SSFP imaging at 1.5 T [2]. Possible explanations are: i) differences in the pulse sequence design, ii) differences in the spatial resolution, with lower resolution being associated with more partial volume and potentially higher T_2_ values, and iii) T_1_ relaxation effects due to higher T_1_ values at 3 T versus 1.5 T. Generally, myocardial T_2_ reported in the literature varies substantially, ranging from about 50 ms to 58 ms at 1.5 T [2]. The heterogeneity of data underlines that the measured T_2_ relaxation time is very sensitive to cofactors and emphasizes the need to generate reference values specific for each technique and imaging setting.

Our results showed that T_2_ increased from base to apex, which is in accordance with a recent work using a similar mapping technique at 1.5 T [15]. The most probable cause is partial-volume effects that increase towards the apex owing to the curvature of the left ventricle. To encounter this limitation, some groups exclude the apical slice from mapping to omit measurement errors [6]. We tried to minimize this error by carefully drawing the contours in the middle of the myocardium while leaving out the endocardial portion of the myocardium, as well as by using an isotropic spatial resolution as high as possible.

Most of the previous studies reported T_2_ values averaged over all myocardial segments or only for a midventricular slice. By averaging T_2_ values over the whole slice or the whole heart, focal T_2_ deviations may be overlooked. The present study is the largest study, which reports T_2_ values for each myocardial segment and slice.

As reported for the global T_2_ values, the segmental T_2_ values increased from base to apex. In comparison, Markl et al. reported T_2_ values from 50.5 ms to 51.6 ms in the basal slice and 54.3 ms to 56.1 ms in the apical slice at 1.5 T [15].

The inter-subject variability of absolute T_2_ values was relatively large both per-slice and per-segment. This finding is in concordance with Thavendiranathan et al., who described T_2_ values ranging from about 50 ms to 62 ms in healthy controls [4], and with Giri et al., who reported that the apical region showed the most pronounced inter-subject variability [2]. The high inter-subject variability can be considered as the main challenge of T_2_ mapping, given that the difference in T_2_ between healthy and injured myocardium has been reported to be relatively small, e.g. 13 ms/11 ms between infarct core/myocarditis and remote myocardium [3,4].

The association of heart rate and T_2_ relaxation time is under discussion. Giri et al. reported that the variability between healthy subjects was unrelated to heart rate. Other studies reported lower T_2_ values in patients with higher heart rate [1,4]. This may be attributed to the hypothesis that higher heart rates induce pronounced T_1_ relaxation effects caused by incomplete T_1_ relaxation, which may affect T_2_ mapping using a SSFP-based approach. This finding is very relevant for clinical practice as subtle T_2_ increases may disappear in acutely ill patients with higher heart rates.

### T_1_ mapping

T_1_ mapping demonstrated diagnostic image quality for the vast majority of myocardial segments. However, a relevant number of myocardial segments had to be excluded due to technical challenges, which would lead to diagnostic uncertainty in a clinical scenario. Previous studies at 1.5 T and 3 T reported lower rates of artifact-related non-diagnostic segments [7,10,16,17]. The explicit source of the artifacts has not been reported in detail in most studies, which renders benchmarking against previous results challenging. A possible contributing factor might be that artifacts are often only visible in the original images - which are used for quality assessment - while they might be not apparent in the final maps. In our study, susceptibility artifacts in the inferolateral region were most frequent.

The pre-contrast T_1_ values are in concordance with Piechnik et al., who reported T_1_ = 1169 ms averaged over all myocardial segments [16]. At 3.0 T higher midventricular T_1_ values (T_1_ = 1315 ms or T_1_ = 1286 ms) were reported when using a T_1_ mapping technique similar to that used in this study [17,18]. These discrepancies underline that T_1_ relaxation times are sensitive to many influencing factors.

The myocardial T_1_ relaxation times reported here can be regarded as reference values specific only for this cohort, time point, mapping technique, type and dosage of contrast media. Further comparisons with other published results are difficult unless an identical study design is used. To provide a context, Lee et al. used 0.15 mmol Gadolinium DTPA and measured a mean T_1_ of about 550 ms in one midventricular slice after 8.5 min in healthy human subjects at 3 T [17].

We observed that the pre-contrast T_1_ times increased from base to apex, whereas the post-contrast T_1_ values decreased from base to apex. Partial-volume effects owing to the curvature of the left ventricle can most probably explain this finding with blood signal being included into the voxel. While some completely exclude apical T_1_ maps from analysis [6], we tried to minimize this error by excluding the endocardial portion of the myocardium and by choosing a high isotropic spatial resolution.

In agreement with Kawel et al. we did not observe significant segment-to-segment differences post-contrast [7]. However, pre-contrast T_1_ values of the anterior segments were lower than T_1_ observed for the other segments. Interestingly, Piechnik et al. observed the identical pattern with MOLLI at 3 T [16]. Kawel et al. confirmed the presence of regional variability of pre-contrast T_1_ values inspite of using a different classification into “septal” and “non-septal” myocardium [7]. Although absolute regional difference was small, this finding has to be considered in clinical CMR interpretation as the difference between healthy and abnormal tissue might be in a similar range.

The inter-subject variability of absolute T_1_ values was notable both per-slice and per-segment, including extreme outliers. This finding is in concordance with other T_1_ mapping studies reporting pre-contrast T_1_ values at 1.5 T ranging from 862 ms to 1105 ms in healthy volunteers [19] and a coefficient of variation of 4.5% (pre-contrast) and 7.0% (post-contrast) [18]. The high inter-subject range may be the main challenge of T_1_ mapping, given that the difference in T_1_ times between healthy and injured myocardium has been reported to be relatively small depending on the underlying disease. Dall’Armellina et al. reported a mean pre-contrast T_1_ value of 1257 ± 97 ms for acutely infarcted segments compared to 1196 ± 56 ms for normal unaffected segments at 3 T [20]. In other myocardial diseases like Fabry’s disease or amyloidosis, pre-contrast T_1_ may already be accurate enough to differentiate cardiac amyloid patients from normals [21].

Post-contrast T_1_ in the present study was even more variable between subjects than pre-contrast T_1_, attributable to the many factors with influence on the contrast kinetics (e.g. patient weight, hematocrit, renal function). Miller et al. recently demonstrated that even though isolated post-contrast T_1_ measurement showed significant within-subject correlation with histological collagen volume fraction, the between-subject correlations were not significant. Hence, isolated post-contrast T_1_ measurement seems to be insufficient for assessing extracellular volume fraction [22].

Aging was found to be associated with decreasing pre-contrast T_1_ values. This is an interesting aspect that may reflect early age-dependent alterations of myocardial texture. Dall’Armellina et al. and Ugander et al. showed that pre-contrast T_1_ times were increased in acute myocardial ischemia [20,23]. Dass et al. reported increase in pre-contrast T_1_ in cardiomyopathies. Hence, the present reduction of pre-contrast T_1_ with age may sound contradictory [24]. In contrast, in a rat model, diffuse myocardial fibrosis was associated with a non-significant trend towards lower pre-contrast T_1_ values [25]. Therefore our data are stimulating to further analyze the value of pre-contrast T_1_ mapping in non-ischemic heart disease in future.

## Conclusion

In conclusion, myocardial T_2_ and T_1_ mapping at 3 T are feasible with a good diagnostic image quality, although susceptibility artifacts related to the magnetic field strength of 3 T triggered exclusion of myocardial segments from analysis. This study provides reference values for myocardial T_2_ and T_1_ relaxation times per slice and per segment for the specific MR setting, which were deduced from a large cohort of healthy volunteers. With this approach a relatively high inter-subject distribution became apparent, which may constitute a relevant challenge for the definition of cut-offs that differentiate healthy from diseased myocardium in clinical practice.

### Study limitations

i) As hematocrit was not measured in this study, its effect on T_2_ and T_1_ relaxation times could not be assessed. ii) Whereas observer variability to assess T_2_ and T_1_ relaxation times was low, the inter-scan variability was not assessed and deserves further investigation. iii) Regarding T_1_ estimation by the applied MOLLI technique, there are known limitations to inversion efficiency [26] and to evaluation of magnitude based data. The inversion efficiency is also dependent on T_2_. At the time the study was designed, an improved inversion pulse designed for myocardial T_1_ mapping tailored for myocardial T_2_ was not available yet. The limitations of evaluating T_1_ based on magnitude images are described in a recent publication [27]. By the time the study was designed, the proposed phase-sensitive recon was not yet available on our system.

## Competing interests

The co-author A. Greiser is employee of Siemens Healthcare, Germany. The other authors declare that they have no competing interests.

## Authors’ contributions

FvKB defined the design of the study, headed its coordination, acquired the image data, read the images, assisted in statistical analysis and drafted the manuscript. MP contributed to the analysis and interpretation of the data and was involved in drafting the manuscript. MD made contributions to acquisition of data and was involved in drafting the manuscript. RW and AG made substantial contributions to the analysis and interpretation of the data and were involved in drafting the manuscript. CS participated in the design of the study, performed the statistical analysis and was involved in drafting the manuscript. TN made substantial contributions to the acquisition, analysis and interpretation of the data and was involved in drafting the manuscript. JSM defined the design of the study, headed its coordination, assisted in statistical analysis and interpretation of the data, and drafted the manuscript. All authors read and approved the final manuscript.

## Supplementary Material

Additional file 1 **T**_**2**_**-mapping.** A full set of T_2_-weighted SSFP single shot images with 3 different T_2_ preparation times and the corresponding T_2_ maps from the basal, midventricular and apical slice. Click here for file

Additional file 2 **T**_**1**_**-mapping.** A full set of T_1_-weighted SSFP single-shot images and the corresponding pre-contrast T_1_ maps from the basal, midventricular and apical slice. Click here for file
