# Supplementary figures and images for: Myocardial T1 and T2 mapping at 3 T: reference values, influencing factors and implications
Source: J Cardiovasc Magn Reson. 2013 Jun 18;15(1):53. doi: 10.1186/1532-429X-15-53 (PMC3702448; doi:10.1186/1532-429X-15-53)

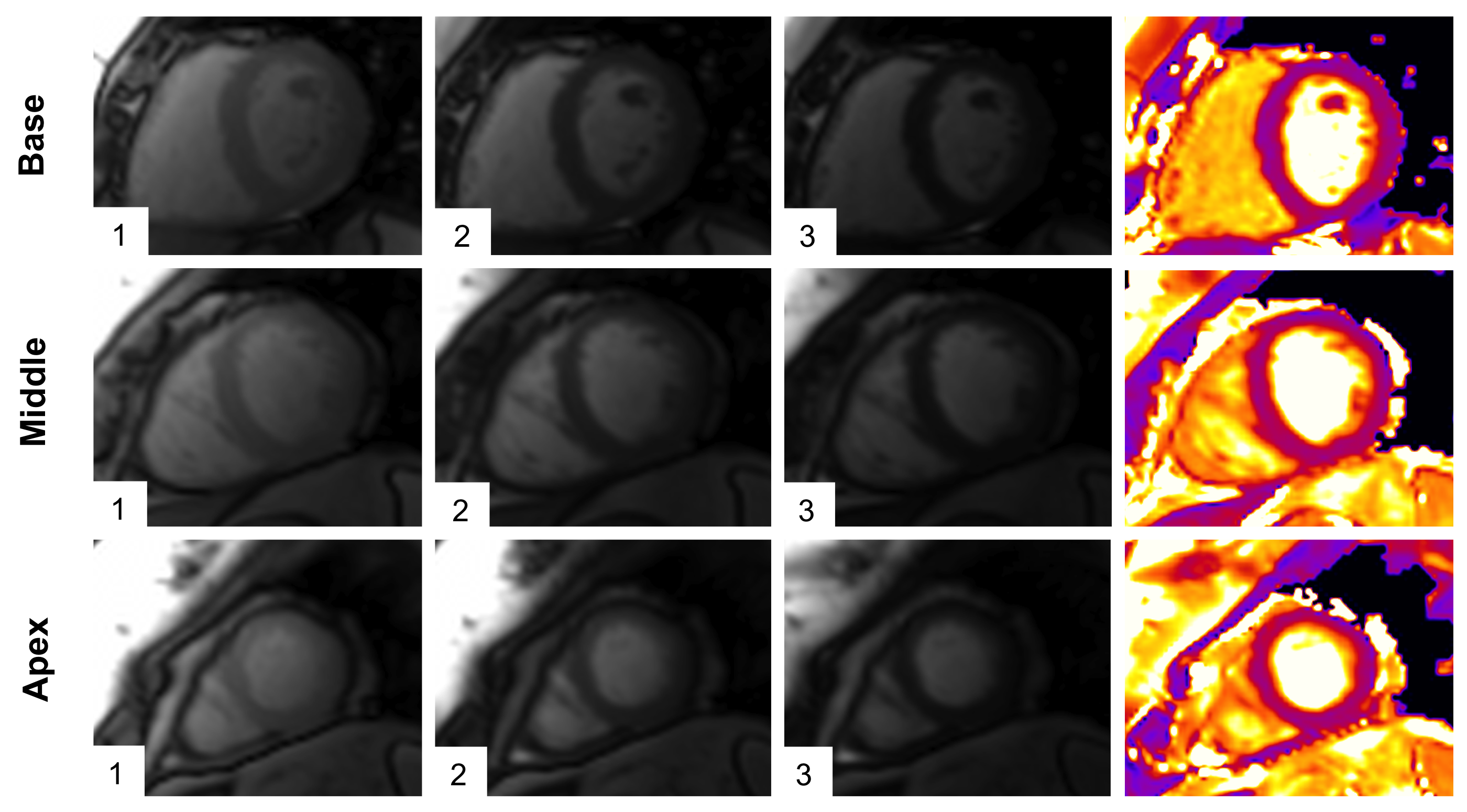

Supplement: Additional file 1 — T2-mapping. A full set of T2-weighted SSFP single shot images with 3 different T2 preparation times and the corresponding T2 maps from the basal, midventricular and apical slice. [file 1532-429X-15-53-S1.tiff]

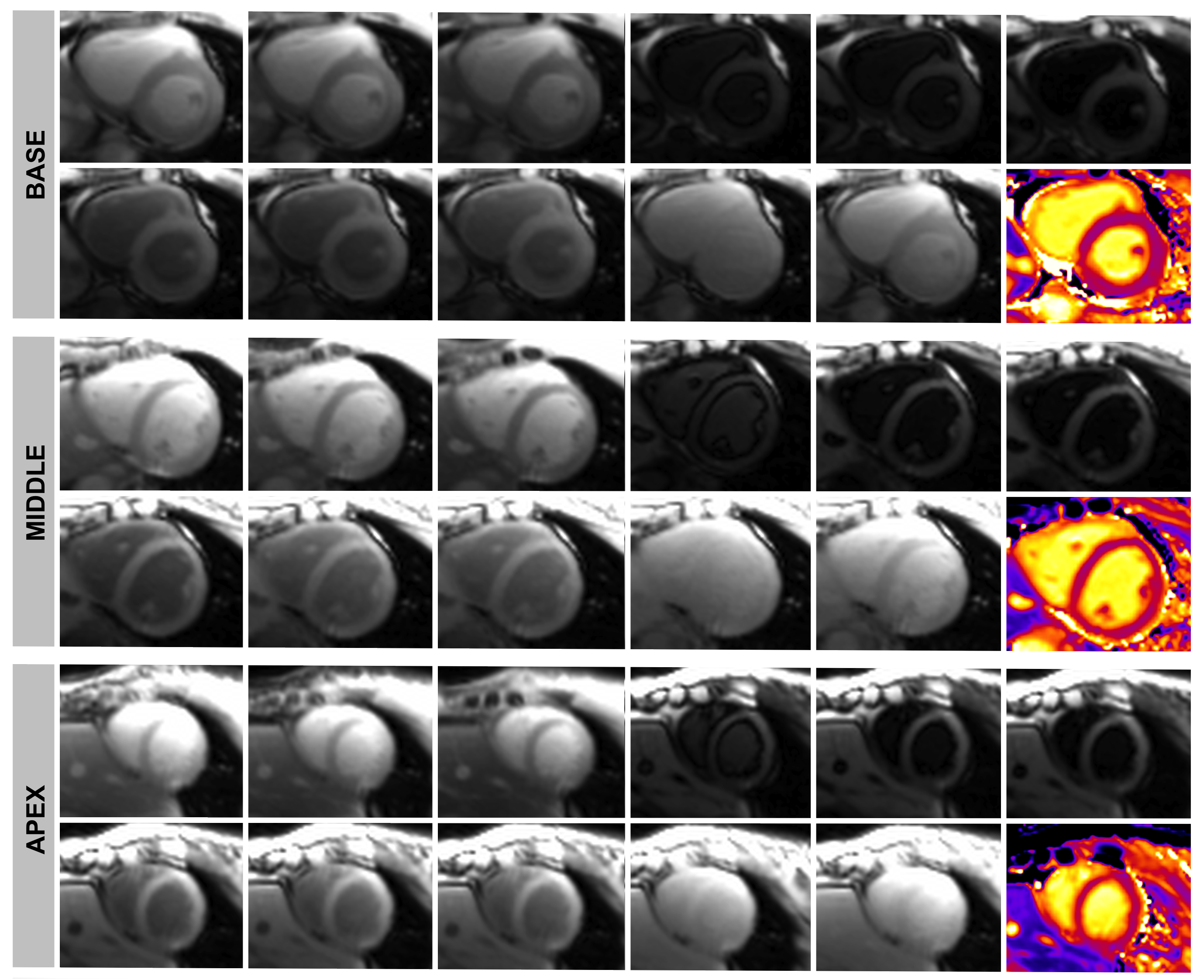

Supplement: Additional file 2 — T1-mapping. A full set of T1-weighted SSFP single-shot images and the corresponding pre-contrast T1 maps from the basal, midventricular and apical slice. [file 1532-429X-15-53-S2.tiff]
